# Nighttime sleep duration and the prevalence of hyperuricemia: a systematic review and network meta-analysis

**DOI:** 10.3389/fnins.2025.1436116

**Published:** 2025-05-09

**Authors:** Chun Luo, FengQi Zhang, Danqian Shen, Jing Sun, YuShan Zhang, ZhiJun Xie, XiaLi Yu

**Affiliations:** ^1^The First School of Clinical Medicine, Zhejiang Chinese Medical University, Hangzhou, China; ^2^School of Basic Medical Sciences, Zhejiang Chinese Medical University, Hangzhou, China; ^3^The Second School of Clinical Medicine, Zhejiang Chinese Medical University, Hangzhou, China; ^4^Department of Orthopedics, Xinchang Hospital Affiliated to Wenzhou Medical University, Xinchang, China; ^5^Department of Rheumatology, The First Affiliated Hospital of Zhejiang Chinese Medical University (Zhejiang Provincial Hospital of Chinese Medicine), Hangzhou, China

**Keywords:** nighttime sleep duration, hyperuricemia, systematic review, network meta-analysis, HUA

## Abstract

**Objective:**

According to clinical observation and recent studies, there is a significant association between night sleep duration and hyperuricemia. In this study, systematic review and network meta-analysis were performed to evaluate the risk of hyperuricemia associated with different nighttime sleep durations.

**Methods:**

Seven databases were searched from database inception to March 2020. Two reviewers independently performed study selection, quality appraisal, and data extraction. Conventional meta-analysis was conducted using either a fixed-effects or random-effects model according to statistical heterogeneity. A Bayesian network meta-analysis was conducted using the consistency model.

**Results:**

Six studies with 416,684 patients and involving different nighttime sleep durations were included. The network meta-analysis showed that compared with normal nighttime sleep duration, the pooled risk ratio (RR) for short nighttime sleep duration was 1.26 (95% confidence interval [CI] 1.22–1.30, *p* < 0.00001). Compared with long nighttime sleep duration, pooled RR of HUA with normal nighttime sleep duration was 0.81 (95% CI 0.67–0.99, *p* = 0.03). Compared with long nighttime sleep duration, pooled RR of HUA with short nighttime sleep duration was 1.07 (95% CI 0.90–1.28, *p* = 0.43).

**Conclusion:**

The evidence in this network meta-analysis illustrates that both short and long sleep duration increased the risk of hyperuricemia, and short sleep duration was more harmful. Further high-quality studies are required to explore the Mechanism of the nighttime sleep duration influencing hyperuricemia.

**Systematic review registration:**

https://www.crd.york.ac.uk/, CRD42024519628.

## 1 Introduction

Hyperuricemia (HUA) is one of the most common metabolic disorders in modern society and the worldwide prevalence has been reported to be 20.1% (Chen-Xu et al., 2019). The human body produces uric acid as the final byproduct of purine metabolism and excessive uric acid levels are the direct cause of gout and gouty arthritis (Yanai et al., 2021).

Sleep is a critical determinant for metabolism, and disturbance of sleep–wake cycles has related to dysregulation of homeostasis. Sleep disorders could activate proteolytic pathways and produce purine, which can break down into uric acid. Short sleep duration has been shown to be correlated with increasing uric acid levels, while poor sleep quality has shown different effects on uric acid (Xiong et al., 2020). Inadequate sleep patterns have been associated with detrimental effects on human well-being. According to the National Sleep Foundation, the optimal sleep duration for adults is 7–8 h (Hirshkowitz et al., 2015). Several studies have shown a U-shaped association between nighttime sleep duration and health conditions such as diabetes (Nôga et al., 2024), hypertension (Scott et al., 2023), cardiovascular disease (Kim et al., 2023), coronary heart disease (Huang et al., 2022), and obesity (Che et al., 2021), and a similar association with all-cause mortality (Huang et al., 2022). These findings suggest that short and long nighttime sleep duration may be risk factors for HUA. In addition, without a dose–response analysis, it remains unknown how many hours of habitual sleep are associated with the lowest risk of HUA. Based on clinical observations and previous studies, we have found that night shift work is associated with increased risk of HUA (Chen et al., 2022; Zhang et al., 2023). As an absolute indicator, prevalence can directly reflect the disease burden in a given population. Therefore, it is critical to public health that the prevalence of HUA among patients according to nighttime sleep duration is summarized. This could help healthcare systems develop appropriate guidance and interventions to prevent and treat HUA in night shift workers.

Network meta-analyses are valued for the high level of evidence they provide, not only in the analysis of multiple risk factors, but also to help identify the most important risk factors (Hoaglin et al., 2011). In this study, we conducted a network meta-analysis of published cross-sectional studies on nocturnal sleep duration in patients with HUA. The purpose of this analysis was to compare the effects of nighttime sleep duration on HUA patients.

## 2 Materials and methods

### 2.1 Search strategy

This systematic review and network meta-analysis followed the Meta-analysis Of Observational Studies in Epidemiology (MOOSE) guidelines. The entire process was performed in accordance with the Preferred Reporting Items for Systematic Reviews and Meta-Analysis (PRISMA) statement (Hutton et al., 2015). The protocol was registered in PROSPERO with number CRD42024519628 (Supplemental File 1).

Two researchers (Luo and Zhang) searched seven databases (PubMed, Cochrane Library, EMBASE, CNKI, WANFANG, CBM, and ClinicalTrials.gov) to find relevant articles published up to 1 March 2020. This study employed English and Chinese as the designated languages. Free terms and MeSH terms, such as (sleep) OR (duration, sleep) OR (total sleep time) OR (sleep quantity) OR (quantity, sleep) OR (sleep quantities) OR (longitudinal sleep) OR (sleep insufficiency) OR (poor sleep) OR (sleep-deprived) OR (sleep problems) OR (sleep disturbances) OR (sleep efficiency) OR (sleep latency) OR (sleep disorders) OR (somnipathy) AND (hyperuricemia) OR (uric acid), were used to search for relevant articles (Supplemental File 2). Further, we conducted a thorough examination of the references of the cited articles to look for any other studies that might be suitable. In addition, to obtain additional study information or supplement missing data, we made the necessary contact with the relevant authors by email. The language of the literature was not limited when selecting the literature for this analysis.

### 2.2 Inclusion and exclusion criteria

Each included study was carefully evaluated by two investigators (Luo and Zhang). When disagreement between the evaluators occurred, a third researcher (Yu) made an impartial ruling based on established protocols. In addition, communicating with the study authors via email to supplement and verify the accuracy of the data was an important step in this study. Any disagreement was resolved through discussion or consultation with two experts (Xie and Yu).

The inclusion criteria were as follows: (1) studies conducted in adults (aged ≥18 years); (2) studies in which the case group was diagnosed with HUA either by imaging or serology; (3) studies in which the control group included healthy individuals without any metabolic diseases; and (4) articles with a focus on the circulating levels of sleep duration.

The exclusion criteria were any of the following: (1) studies of patients without HUA or with HUA with other metabolic diseases; (2) studies reporting factors associated with secondary uric acid intake or metabolism, such as alcohol consumption, use of harmful drugs, genetic disorders, or other types of kidney disease; (3) studies with other similar indices but not sleep duration; (4) study reports of sleep duration in the night, not at daytime; (5) studies with non-comparison with healthy individuals; (6) case reports, review literature, or animal experimental research; (7) repetitive articles; and (8) articles missing important data and with no reply from the corresponding author.

### 2.3 Literature selection and data extraction

To verify the accuracy of the data, data extraction was performed independently by two investigators. At the same time, these two reviewers independently collected key study information using pre-set standardized data extraction forms. The main data were first author, publication year, research site (country), number of subjects, sex (male %), mean age of the study population, sleep duration categories, study design, covariates used in adjustment, and study quality.

### 2.4 Risk of bias

The risk of bias of the included studies was assessed by two independent reviewers using the revised version of the Agency for Healthcare Research and Quality (AHRQ) for cross-sectional studies (Atkins et al., 2005). Disagreements were resolved either by consensus or by a third reviewer. Finally, the overall bias of studies was identified. Studies with a score of 8 points or higher were considered as high quality; between 4 and 7 points were considered as moderate quality; and 3 points or lower were considered as low quality.

### 2.5 Statistical analysis

First, we used Review Manager Version 5.4 to perform a conventional pairwise meta-analysis. For the dichotomous variable, risk ratio (RR) with 95% confidence interval (CI) was used to estimate the effect size. Statistical heterogeneity between eligible studies was evaluated using Cochran’s *Q* test (Higgins et al., 2003) and *I*^2^ test (Chen and Benedetti, 2017). If the data were not significantly heterogeneous (*Q* test with *p*-value > 0.10 and *I*^2^ ≤ 50%), we chose a fixed-effects model to estimate the effect size. Otherwise, a random-effects model was applied.

Second, Stata 15.1 software (including network analysis and network graphics module) was used to perform network meta-analysis in a Bayesian framework. Network meta-analysis extends principles of conventional meta-analysis to evaluate multiple risk factors in a single analysis, which is achieved by combining both indirect evidence from comparisons of different interventions against a common compactor and direct evidence reported in head-to-head trials. When there was no direct evidence between two risk factors, the result was only from indirect evidence. Network diagrams were drawn to show the comparative relationships between the different interventions. We combined all individual study data from any pair of risk factors to estimate pooled effect sizes, which were presented as RRs with their 95% confidence intervals (95% CI). Instead of ORs, RRs were used to aid comparability across studies. In the context of this particular study, where distinct incidence rates are ascertainable, employing the RRs as the measure of effect size more accurately captures the disparities in risk between the compared groups. Furthermore, under conditions of low overall incidence, the ORs and RRs can be deemed to be reasonably equivalent, providing a reliable approximation for comparative analysis.

The inconsistency of individual studies was evaluated by analysis of inconsistency factors and node-splitting analysis. When the inconsistency factor contained a neutral value (0) and node-splitting analysis showed *p*-value ≥ 0.05, the consistency model was applied as there was no significant inconsistency between studies. Otherwise, the inconsistency model would be used. The convergence of the model was evaluated by the latent Potential Scale Reduction Factor (PSRF). The closer this index is to 1, the more ideal the convergence of the model is.

To evaluate the association between nocturnal sleep duration and the risk of HUA, we used the surface under the cumulative ranking curve (SUCRA) as a metric. SUCRA values range from 0 to 100%, with 0% indicating the highest risk and 100% indicating the lowest risk. Ranking probabilities were also estimated and the number of iterations of the Markov chain was calculated to evaluate the degree of convergence of the model. At the same time, a corrected funnel plot was constructed to systematically evaluate the possible publication bias (Egger et al., 1997).

## 3 Results

### 3.1 Characteristics of the studies

**Figure 1:**
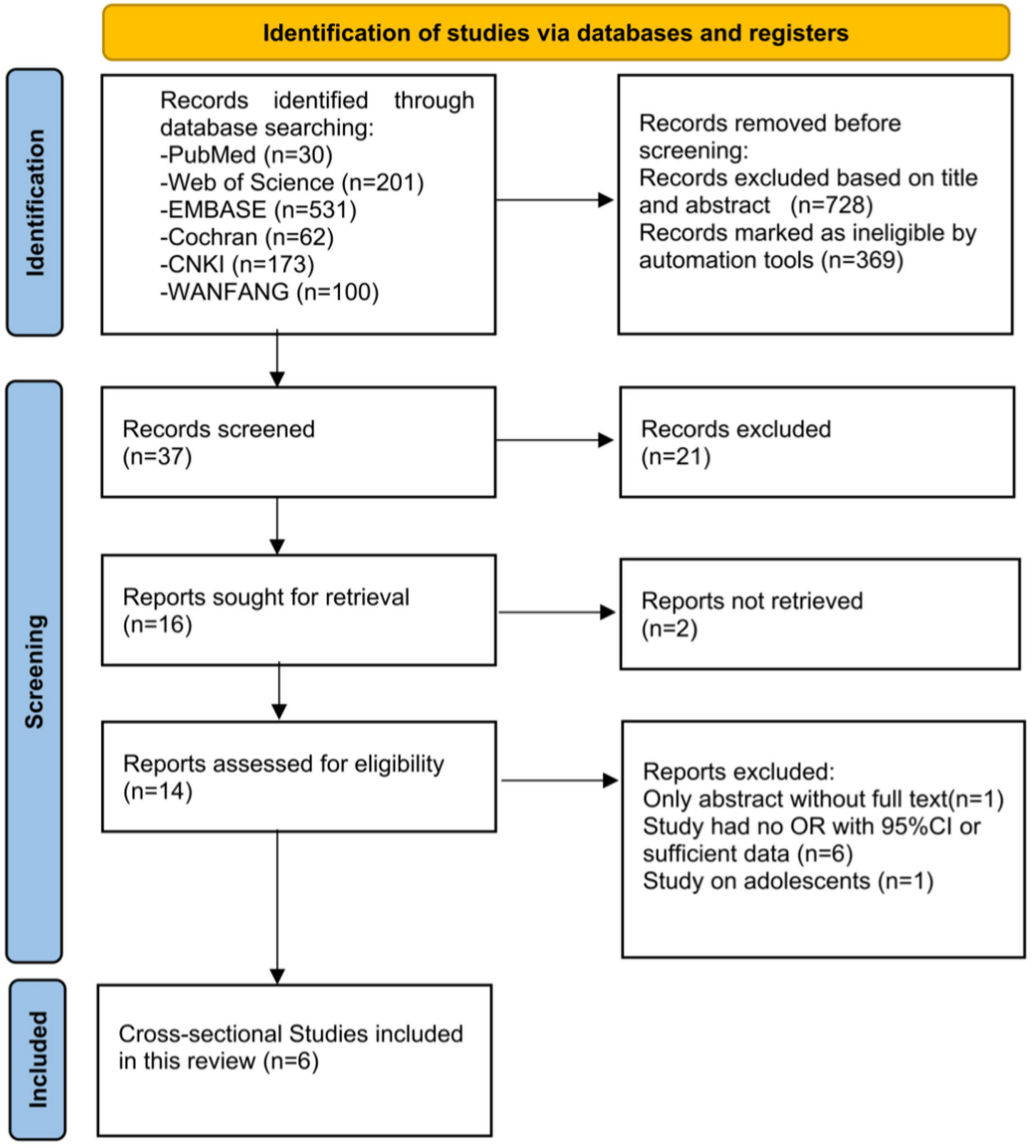
Flow diagram showing the study selection process.

The overall process is displayed in the form of a PRISMA 2020 flow diagram in Figure 1. A total of 1,097 articles and abstracts were identified through the search strategy. After initial screening, 1,083 duplicates and ineligible articles were excluded. The titles and abstracts of the remaining articles were read, and 14 studies were identified as eligible. After reviewing the full texts of the 14 articles, 8 articles were excluded. Finally, six cross-sectional studies (Saito et al., 2022; Noh et al., 2017; An et al., 2022; Zou et al., 2022; Yu et al., 2021; Yu et al., 2020) on the prevalence of HUA among patients with various nighttime sleep durations were included for analysis. In total, there were four studies performed in China, one in Japan, and one in the United Kingdom (UK).

Nighttime sleep duration categories (hours) and other characteristics of the included studies are presented in Table 1. A total of 416,684 patients with various nighttime sleep durations were enrolled in the included studies. The median age of the patients ranged from 36.8 to 71.2 years. The percentage of males ranged from 32.9 to 59.8%. The year of publication of the studies ranged from 2020 to 2024. The overall quality of the studies was relatively reliable. The results of subgroup analysis are shown in the Table 2. The proportion of males and mean age did not significantly explain the heterogeneity between studies (*P* > 0.05). Neither sex nor age distribution was significantly associated with hyperuricemia.

**Table 1:** Baseline characteristics of studies included in the meta-analysis.

Author, year	Country	Participants (n)	Cases (n)	Sex (male %)	Mean age, years (range)	Comparison categories (hours)	Study design	Adjustment	Quality of study (AHRQ points)
An et al. (2022)	Henan, CHINA	10,321	1,775	32.9	36.8 (20–60)	Short: <7 Normal: 7–8 Long: >8	Cross-sectional	1, 2, 3, 7, 8, 9, 11, 13	8
Zou et al. (2022)	UK	387,980	25,846	46.0	56.9 (±7.8)	Short: <5 Normal: 7 Long: ≥9	Cross-sectional	1, 2, 4, 5, 12, 13	10
Yu et al. (2021)	CHINA	8,289	1,302	52.8	56.5 (≥18)	Short: 5–6 Normal: 7–8 Long: 9–10	Cross-sectional	1, 2, 3, 6, 13	9
Yu et al. (2020)	CHINA	1,744	227	50.7	71.2 (±4.9)	Short: ≤7 Normal: 7–8 Long: >8	Cross-sectional	1, 2, 4, 6, 8, 9, 10, 13	8
Saito et al. (2022)	JAPAN	2,538	311	59.8	56.4 (±10.8)	Short: <6 Normal: 6–8 Long: ≥8	Cross-sectional	1, 2, 4, 8, 9, 13	9
Zhenyu et al. (2022)	Beijing, CHINA	5,812	1,175	45.2	46.9 (±16.0)	Short: <7 Normal: 7–9 Long: >9	Cross-sectional	1, 2, 4, 7, 9, 10, 11, 13	9

Adjustment: 1, age; 2, sex; 3, education; 4, marital status; 5, residential remoteness; 6, alcohol consumption; 7, smoking status; 8, health insurance status; 9, employment status; 10, income; 11, body mass index (BMI); 12, physical activity; 13, baseline health status. Ref: Reference normal nighttime sleep duration.

**Table 2:** Subgroup analysis of nighttime sleep.

Characteristics	Study (n)	RR (95%CI)	*I*^2^ (%)	*P_h_*
**Short sleep**				
Sex (male %)				
<0.5	3	1.13 (0.96–1.30)	72	0.09
≥0.5	3	1.11 (1.00–1.22)	43	0.24
Mean age (years)				
<50	2	1.15 (1.05–1.25)	0	0.49
>50	4	1.13 (0.96–1.37)	67	0.03
**Long sleep**				
Sex (male %)				
<0.5	3	1.08 (0.91–1.29)	65	0.01
≥0.5	3	1.03 (0.87–1.26)	62	0.03
Mean age (years)				
<50	2	1.18 (0.95–1.46)	55	0.07
>50	4	0.99 (0.90–1.08)	0	0.63

*P_h_*: *P*-value of the within-group heterogeneity test based on the Cochran’s Q test.

### 3.2 Risk of nighttime sleep duration by conventional pairwise meta-analysis

**Figure 2:**
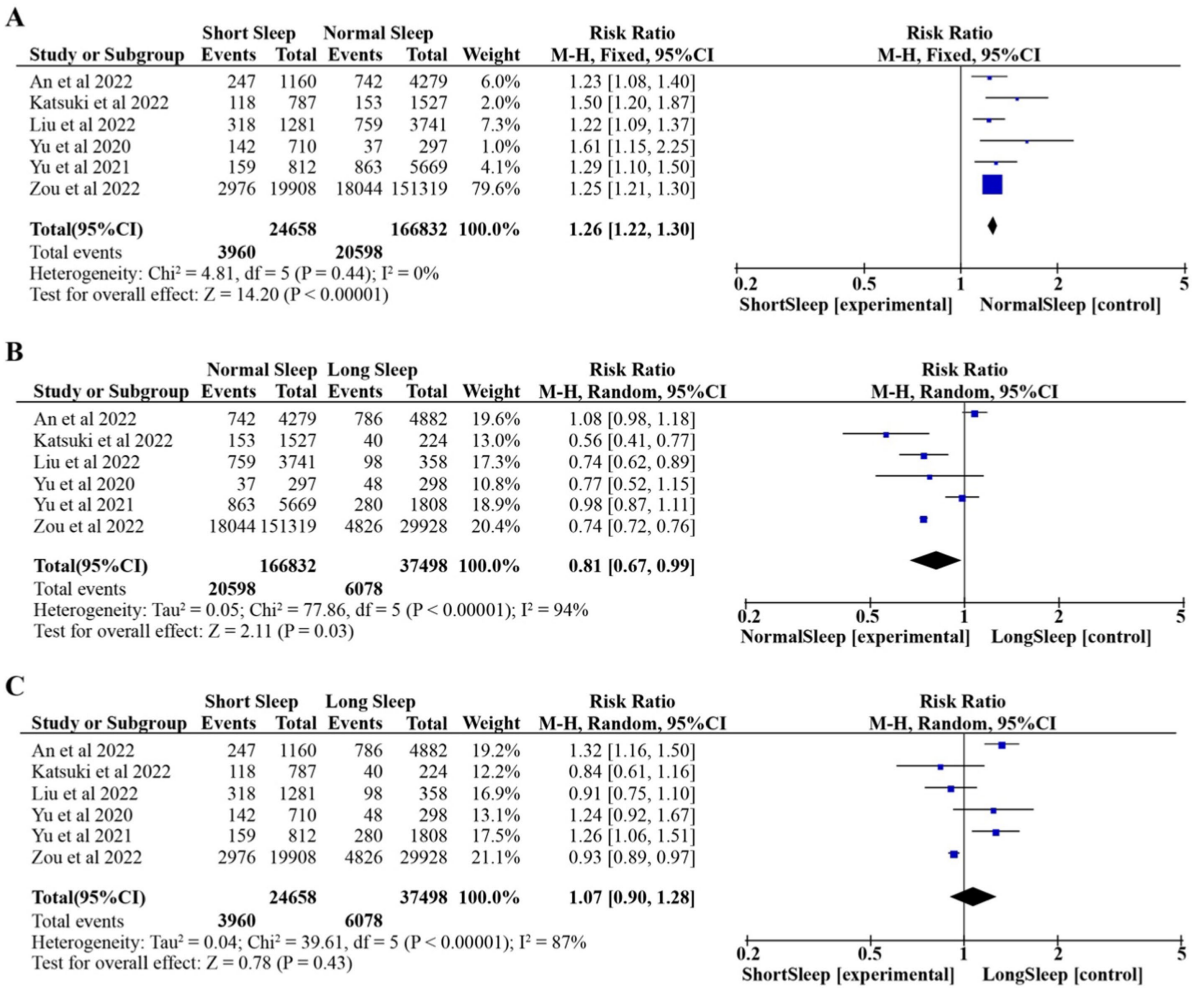
Pooled effect of different nighttime sleep duration on HUA. Forest plots for each nighttime sleep duration. **(A)** Short Sleep vs. Normal Sleep; **(B)** Long Sleep vs. Normal Sleep; **(C)** Short Sleep vs. Long Sleep.

The results of conventional pairwise meta-analysis of nighttime sleep duration and HUA are reported in this section. The fixed-effects model was used because there was no heterogeneity among the studies (*p* = 0.44, *I*^2^ = 0%). As shown in Figure 2A, compared with normal nighttime sleep duration, Short nighttime sleep duration resulted in a increase in the risk of HUA (RR:1.26, 95% CI:1.22–1.30, *p* < 0.00001).

The random-effects model was used because of the high heterogeneity among the studies (*p* < 0.00001, *I*^2^ = 94%). As shown in Figure 2B, compared with long nighttime sleep duration, normal nighttime sleep duration resulted in a reduction in the risk of HUA (RR: 0.81, 95% CI: 0.67–0.99, *p* = 0.03).

The random-effects model was used because of the high heterogeneity among the studies (*p* < 0.00001, *I*^2^ = 87%). As shown in Figure 2C, compared with long nighttime sleep duration, short nighttime sleep duration resulted in increase in the risk of HUA (RR:1.07, 95% CI:0.90–1.28, *p* = 0.43).

### 3.3 Risk of HUA according to nighttime sleep duration by Bayesian network meta-analysis

**Figure 3:**
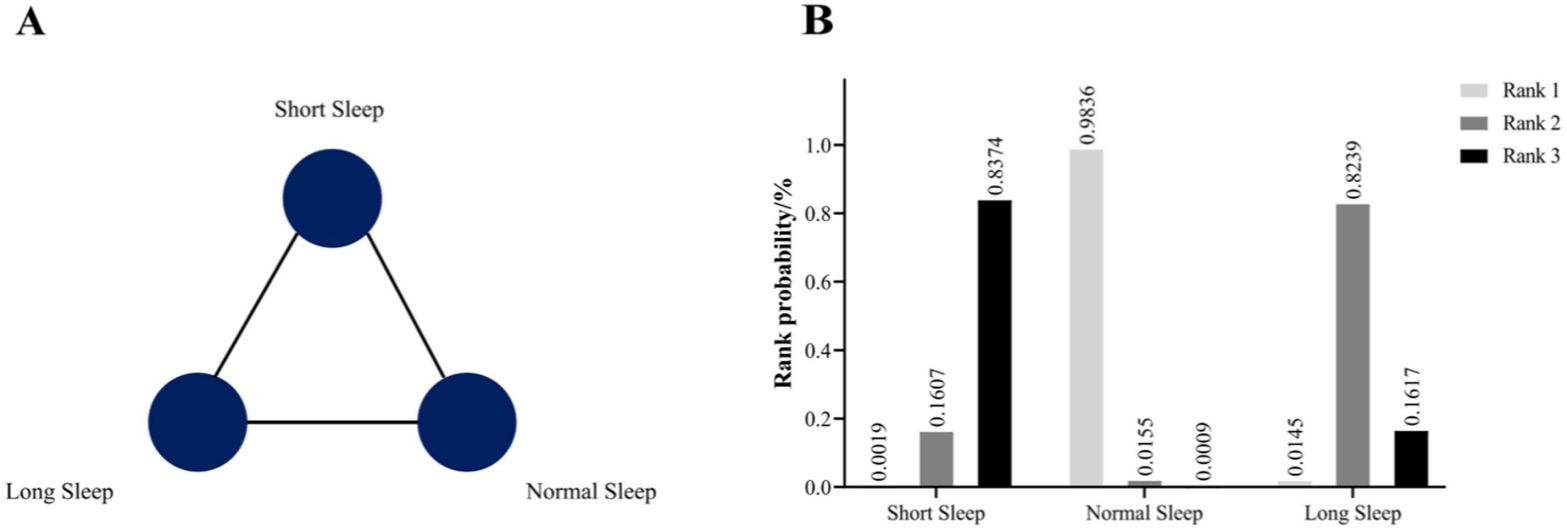
**(A)** Prevalence evidence network map of three kinds of nighttime sleep durations. **(B)** The rank probability of the efficacy of three different nighttime sleep durations on HUA risk.

The results of the inconsistency model showed that the neutral values were contained in the 95% CI of the inconsistency factor, and node-splitting analysis showed *p* values were ≥0.05, so the consistency model was applied to the Bayesian network meta-analysis. The PSRF value ranged from 1.00–1.011, indicating that the consistency model had relatively better convergence. Figure 3A presents the network map for nighttime sleep duration, showing the three kinds of comparison that were frequently used and connected closely. Table 3 shows the relative effects of three kinds of nighttime sleep durations by RRs with their 95%CIs. Compared with short nighttime sleep duration, normal nighttime sleep duration had significant positive effects on HUA risk (RR = 0.76, 95% CI 0.64–0.90). When compared with normal nighttime sleep duration, long nighttime sleep duration had significant negative effects on HUA (RR = 1.21, 95% CI 1.02–1.45). Additionally, compared with short nighttime sleep duration, longer nighttime sleep duration may benefit HUA prevention (RR = 0.92, 95% CI 0.78–1.10). In this study, we employed Stata software to determine the likelihood that the three distinct nocturnal sleep durations were assigned the top three ranks, respectively. Subsequently, these probabilities were graphically represented as a “time-rank” probability bar chart (Figure 3B). This visualization technique was employed to provide a more precise depiction of the influence exerted by varying nocturnal sleep durations on the development of hyperuricemia. The results revealed that normal nighttime sleep had the highest probability to be ranked as first (98.36%), followed by long sleep (ranked second; 82.39%) and short sleep (ranked third; 83.74%) duration. According to this single ranking results, the prevalence of HUA in normal sleep may be the lowest, followed by long sleep, and finally short sleep.

**Table 3:** Relative effects of different nighttime sleep durations on HUA risk.

**Long sleep**		
1.21 (1.02, 1.45)	**Normal sleep**	
0.92 (0.78, 1.10)	0.76 (0.64, 0.90)	**Short sleep**

### 3.4 Publication bias

**Figure 4:**
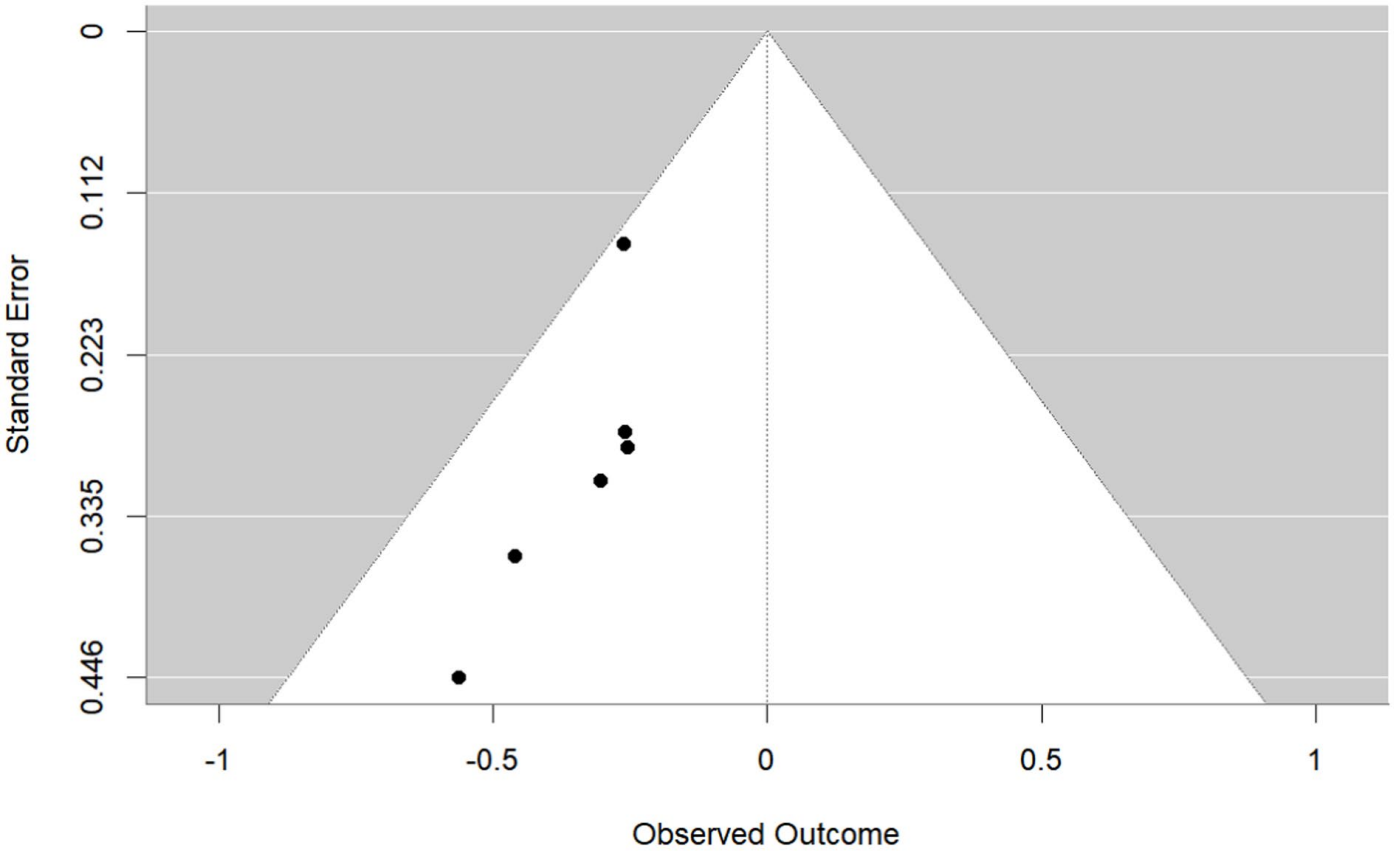
Funnel plot.

**Figure 5:**
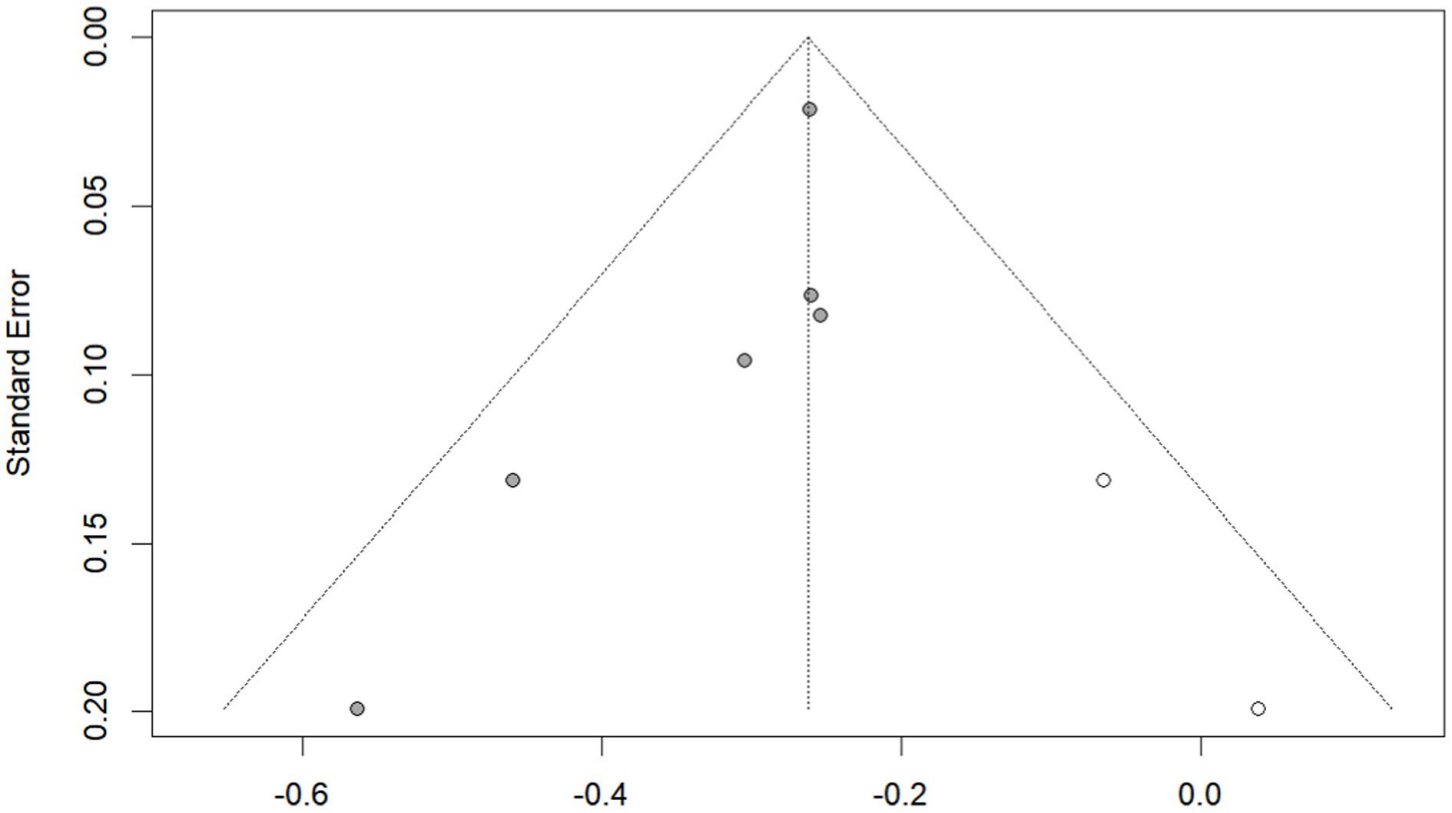
Funnel plot after trim-and-fill correction procedure.

Publication bias was investigated using the funnel plot method. By observation, the shape of the funnel plot (Figure 4) showed evident asymmetry, suggesting a possibility of publication bias. And Egger’s regression test results (*p* = 0.029 < 0.05) indicated publication bias. We then performed the trim-and-fill correction procedure (Figure 5) by Stata15.1 software. Following the incorporation of two additional virtual documents subsequent to the iteration process, the meta-analytic outcomes remained statistically inalterable (*p* < 0.05), thereby demonstrating the robustness of our findings.

## 4 Discussion

We conducted a comprehensive search of relevant publications and integrated the available evidence from six cross-sectional studies with a total of 416,684 participants examining the association of nighttime sleep duration and prevalent HUA. The main findings were: (1) that a U-shaped relationship existed between habitual self-reported nighttime sleep duration and HUA, and (2) sleep duration that is either shorter or longer than normal, especially shorter hours, is associated with increased prevalence of HUA.

Both conventional meta-analysis and network meta-analysis revealed that both short and long nighttime sleep duration increased risk for HUA compared with normal nighttime sleep duration. In addition, rank probability analysis indicated that short nighttime sleep duration had the highest probability of being rank 1 (84.74%), meaning that short nighttime sleep duration is associated with higher risk for HUA.

### 4.1 Sleep duration in relation to other metabolic disorders

Several meta-analyses have reported a significant association between short sleep duration and sleep duration may affect the activity of growth hormone, cortisol, and even the hypothalamic–pituitary-thyroid (hPT) axis in hyperlipidemia, leading to lipid metabolism disorders and hyperlipidemia (Zhang et al., 2022), Sleep restriction is associated with changes in energy homeostasis, insulin resistance, and β-cell function, which lead to diabetes (Antza et al., 2021), and When sleep is restricted, but diet is controlled at the energy balance required for a typical day of adequate sleep, ghrelin, a hormone that stimulates appetite, is increased, and leptin, a satiety hormone, is decreased, resulting in increased levels of hunger, which leads to increased adiposity (Chaput et al., 2023).

### 4.2 Short sleep duration and HUA

Our findings suggested that short nighttime sleep duration is associated with higher risk for HUA in a dose–response manner. This finding is similar to most previous studies of the relationship between nighttime sleep duration and metabolic diseases. Since these are all metabolic disorders of the human body, it is unsurprising that short sleep duration is associated with disturbances in blood uric acid metabolism.

A number of pathophysiological mechanisms may be associated with short sleep duration and HUA. There is a strong correlation between nocturnal sleep and the regulation of cortisol in humans (Liu and Reddy, 2022). Levels of catecholamines and cortisol can be modulated during sleep, which can subsequently impact uric acid levels (Gil-Lozano et al., 2016). Nighttime sleep deprivation disrupts the balance of melatonin and cortisol secretion. Consequently, nocturnal sleep duration may play a role in epigenetic modifications of metabolic genes and DNA methylation in leukocytes (Jansen et al., 2019). Alterations in sleep duration may lead to aberrant DNA methylation patterns that affect uric acid metabolism (Shi et al., 2023). Behavioral and lifestyle factors appear to partially explain the association between self-reported short sleep duration and HUA (Yokose et al., 2021). For instance, individuals who reported sleeping for less than 6 h were more likely to engage in health-risk behaviors, including smoking and excessive alcohol consumption, to engage in less physical activity during leisure time, and to consume fewer fruits and vegetables. These health-risk behaviors have been associated with an increased risk of HUA.

### 4.3 Long sleep duration and HUA

Longer nighttime sleep duration, compared with normal nighttime sleep duration, is associated with increased risks for multiple health issues, including diabetes, obesity (Tan et al., 2018), stroke, coronary artery disease, cardiovascular disease (Yin et al., 2017), and death from all causes. In addition, similar to short sleep duration, a positive dose–response relationship was found between long sleep duration and the prevalence of HUA, which led to a significant increase in RRs of prevalence.

The potential association between increased sleep duration and HUA may involve multiple mechanisms. Studies have shown that longer sleep duration is associated with sleep disruption, often caused by extended periods of bed rest, as a potential cause of daytime sleepiness and fatigue (Zamore and Veasey, 2022). Sleep disruption and the resulting daytime sleepiness and fatigue are associated with chronic inflammation and elevated cortisol levels, all of which are thought to be risk factors for metabolic diseases including HUA (De Leeuw et al., 2023). In addition, increased sleep duration may cause dysregulation of uric acid levels by changing the composition of gut microbiota and affecting the metabolism of purine nucleotides, precursors of uric acid (Wang et al., 2022). Prolonged sleep duration may also disrupt the circadian rhythm regulated by the brain, which is particularly common in shift workers (Boivin et al., 2022). It may promote the production of inflammatory factors such as IL-6 and TNF-*α*, which may lead to renal dysfunction, including glomerular endothelial cell damage, vascular smooth muscle cell proliferation, andRenin-Angiotensin-Aldosterone System (RAAS) activation (Mironova, 2023; Zhang et al., 2023). It ultimately affects the ability of the kidney to metabolize uric acid.

### 4.4 Short and long nighttime sleep duration

According to our network meta-analysis, short nighttime sleep may be more harmful than long sleep. Therefore, other possible explanations should be considered when investigating the association between sleep duration and HUA. In addition to sleep duration per se, studies suggest that self-reported sleep duration may also be related to sleep quality, emotional stress, and sociodemographic factors such as income, labor intensity, ethnicity, and behavioral risk factors (Son et al., 2023; Dehlin et al., 2020). Most of the studies included in the meta-analysis did not consider these possible confounding variables. Therefore, it becomes challenging to accurately assess the specific effects of self-reported short sleep duration, sleep quality, emotional stress, and sociodemographic risk factors on new-onset HUA. Future studies should focus on adjusting for the effect of sleep duration on HUA and explore the associations between changes in sleep duration, emotional stress level, and sleep quality over time and socio-demographic risk factors.

## 5 Study strengths and limitations

To our knowledge, this is the first study that comprehensively integrated the available evidence of the relationship between different nighttime sleep duration and HUA. This study conducted a comprehensive analysis of the literature published in the past 5 years that explored the association between nighttime sleep duration and HUA. By integrating multi-dimensional information, we aim to comprehensively understand the association between nighttime sleep duration and HUA. Although a high-purine diet is considered to be the main cause of HUA (Danve et al., 2021), many patients who deny such a diet have abnormal nocturnal sleep duration, including insufficient or excessive sleep. The results of this study help to identify this problem and reveal this risk factor for patients in advance, which may promote the rational conservation and utilization of medical resources.

Although this study provides valuable insights into the association between sleep duration and HUA, several limitations can be identified. First, the small number of articles included limits the strength of evidence for our conclusions. Although other studies have investigated the association between sleep and HUA, most of these studies also considered other sleep disorders, such as sleep apnea syndromes (Zhang et al., 2015). To minimize the interference of confounding variables, we focused on nighttime sleep duration in this study, resulting in a limited total of six included studies. The findings are therefore based on a limited sample size and may not be comprehensive. In addition, the robustness of the findings may be affected by the inclusion of only studies with a cross-sectional design. Second, the majority of the included studies (five of six) were from Asia, with only one study, by Zou et al., from Europe, making the generalizability of the findings uncertain. Although four English language databases were searched, research into HUA is lacking compared with other metabolic diseases. More data sets across regions are needed for comprehensive research conclusions to be reached. Third, the results of our network meta-analysis are based on self-reported sleep duration. Generally, self-reported sleep duration is approximately an hour longer than sleep duration measured objectively using techniques such as polysomnography and actigraphy, and reflects a combination of factors. Self-reported sleep duration not only reflects actual sleep duration but also correlates with other factors, including demographic characteristics, socioeconomic status, educational background, race, risk behaviors (e.g., smoking and heavy drinking), sleep quality, physical health problems (e.g., pain and obesity), and mental health problems (e.g., depression and emotional stress). Finally, the findings showed some heterogeneity, which may be related to variation in study settings, time constraints, ethnicity, and other factors. There may have also been unrecognized sources of bias in the study. Although most studies are tightly controlled for variables such as age and sex, unanticipated confounders may still influence results.

## 6 Conclusion

Providing conclusive evidence on the development of HUA will improve the quality of medical services for patients with this condition. The results suggest that, relative to conventional sleep duration, short or long sleep duration may increase the risk of HUA. Furthermore, the potential negative effect of short sleep duration on HUA appeared to be stronger than that of long sleep duration. We suggest that this area is explored in greater depth in subsequent studies, to accumulate sufficient evidence to guide actual medical practice. Given that these comparisons were based on a limited number of studies and the low level of evidence, future network meta-analyses should include more clinical trials to enhance confidence in the existing findings and generalizations from them.

## Data Availability

The raw data supporting the conclusions of this article will be made available by the authors, without undue reservation.
