# Supplementary material for: Nighttime sleep duration and the prevalence of hyperuricemia: a systematic review and network meta-analysis
Source: Front Neurosci. 2025 May 9;19:1436116. doi: 10.3389/fnins.2025.1436116 (PMC12098448; doi:10.3389/fnins.2025.1436116)
Supplement: Supplementary file 1 [file Supplementary_file_1.pdf]

Nighttime Sleep Duration and the Prevalence of Hyperuricemia: A Systematic Review and Network Meta-Analysis

LUO Chun, YU Xiali, ZHANG Fengqi, SHEN Danqian, XIE Zhijun, Sun Jing

Review methods were amended after registration. Please see the revision notes and previous versions for detail.
To enable PROSPERO to focus on COVID-19 submissions, this registration record has undergone basic automated checks for eligibility and is published exactly as submitted. PROSPERO has never provided peer review, and usual checking by the PROSPERO team does not endorse content. Therefore, automatically published records should be treated as any other PROSPERO registration. Further detail is provided here.

Citation
LUO Chun, YU Xiali, ZHANG Fengqi, SHEN Danqian, XIE Zhijun, Sun Jing. Nighttime Sleep Duration and the Prevalence of Hyperuricemia: A Systematic Review and Network Meta-Analysis. PROSPERO 2024 CRD42024519628. Available from https://www.crd.york.ac.uk/PROSPERO/view/CRD42024519628.

PREVIEW

REVIEW TITLE AND BASIC DETAILS

Review title
Nighttime Sleep Duration and the Prevalence of Hyperuricemia: A Systematic Review and Network Meta-Analysis

Original language title
English

Review objectives
Nighttime sleep is closely related to the body's metabolism and may be a key factor in metabolic syndrome. Hyperuricemia is based on dysfunction of uric acid metabolism. Sleep duration is an important factor in the assessment of sleep quality, and there may be a certain correlation between sleep duration and hyperuricemia, but the specific correlation is still unclear. This study aims to explore whether sleep duration is one of the high-risk factors for hyperuricemia, which will play a role in regulating rest and rest and reducing the incidence of hyperuricemia in the future.

Keywords
nighttime sleep duration; Hyperuricemia; Systematic review; Network meta-analysis; HUA

SEARCHING AND SCREENING

Searches
Two researchers searched seven databases (PubMed, Cochrane Library, EMBASE, CNKI, WANFANG, CBM, and ClinicalTrials.gov) to find relevant articles publishedup to 2 April 2021. Free terms and MeSH terms, were used to search for the relevant articles. In addition, the reference lists of articles with citations were also reviewed to identify any suitable papers. It was also necessary to contact authors by e-mail to clarify additional studies or ask for missing data. There were no language restrictions for the articles selected for the analysis.

Study design
Both randomized and nonrandomized study types will be included.

Included
The inclusion criteria were as follows: 1) conducted in adults (aged ≥18 years); 2) the case group must be diagnosed with Hyperuricemia either by imaging or biopsy； 3) the control group must include healthy individuals without any metabolic diseases; 4) articles that focused on the circulating levels of sleep; and 5) case-control studies or cohort

studies.The exclusion criteria were as follows: 1) patients without Hyperuricemia or Hyperuricemia with other metabolic diseases; 2) factors associated with secondary uric acid intake or metabolism, such as alcohol consumption, use of harmful drugs, genetic disorders, or other types of kidney disease;; 3) other similar indices but not sleep duration 4) sleep duration in the night, not in the daytime ;5) non-comparison with healthy individuals; 6) case report, review literature, or animal experimental research; 7) repetitive articles; and 8) articles missing important data and no reply from the corresponding author.

Excluded
A total of 1,097 articles and abstracts were identified through the search strategy. After initial screening, 1,083 duplicates and ineligible articles were excluded. The titles and abstracts of the remaining articles were read, and 14 studies were identified as

eligible. After reviewing the full texts of the 14 articles, 8 articles were excluded. Finally, six cross-sectional studies

Link to search strategy
A full search strategy has been uploaded to PROSPERO. The PDF may be accessed through this link https://www.crd.york.ac.uk/PROSPEROFILES/dbeb136039ece7adb1b0f0e339f86947.pdf.

ELIGIBILITY CRITERIA

Condition or domain being studied
Hyperuricemia is one of the most common metabolic disorders in modern society. Due to physiological or pathological changes in the body, uric acid, the end product of purine metabolism in the human body, appears metabolic imbalance and accumulation, resulting in elevated blood uric acid, and may lead to some diseases related to crystal deposition, such as gout, gouty nephropathy and urolithiasis [2]. The prevalence of hyperuricemia is about 20% in the world. Currently, there is no national epidemiological data on hyperuricemia, and the prevalence is different in different regions. The prevalence rate in economically developed coastal areas is higher than that in other regions, and the incidence is increasing year by year. Sleep is an essential physiological process of the human body, sleep can help the body to achieve self-repair, adjust the body's metabolic level, so as to maintain good health. Sleep problems increase the risk of hyperuricemia, hyperinsulinemia, hypertension, and cardiovascular disease.

Population

Included
The inclusion criteria were as follows: 1) conducted in adults (aged ≥18 years); 2) the case group must be diagnosed with Hyperuricemia either by imaging or biopsy；3) the control group must include healthy individuals without any metabolic diseases; 4) articles that focused on the circulating levels of sleep; and 5) case-control studies or cohort studies.The exclusion criteria were as follows: 1) patients without Hyperuricemia or Hyperuricemia with other metabolic diseases; 2) factors associated with secondary uric acid intake or metabolism, such as alcohol consumption, use of harmful drugs, genetic disorders, or other types of kidney disease;; 3) other similar indices but not sleep duration 4) sleep duration in the night, not in the daytime ;5) non-comparison with healthy individuals; 6) case report, review literature, or animal experimental research; 7) repetitive articles; and 8) articles missing important data and no reply from the corresponding author.

Intervention(s) or exposure(s)

Included
Different level of sleep duration；Whether you smoke or not；Whether you drink Alcohol or not；Whether to use sleeping medication

Comparator(s) or control(s)

Included
A group of hospital in-patient or healthy people who have enough sleep

Context
To verify the accuracy of the data, data extraction was performed

independently by two investigators. At the same time, these two reviewers independently collected key study information using pre-set standardized data extraction forms. The main data were first author, publication year, research site (country), number of subjects, sex (male %), mean age of the study population, sleep duration categories, study design, covariates used in adjustment, and study quality

OUTCOMES TO BE ANALYSED

Main outcomes

1)Change in Uric acid level from baseline to the last available follow-up;2) Body Mass Index (BMI)

Additional outcomes

Functonal outcomes:estimated glomerular filtration rate (eGFR) decline.

DATA COLLECTION PROCESS

Data extraction (selection and coding)

wo researchers independently extracted data, including the first author's last name, publication date, country of origin, Newcastle–Ottawa Scale (NOS) score, Uric acid level measuring method, numbers of cases and controls, basic information of cases and controls (such as age and sex), diagnostic methods, adjusted factors, levels of Uric acid in the Hyperuricemia group/control group, and Body Mass Index (BMI) in the Hyperuricemia group/control group. In addition, the grading of recommendation, as- sessment, development, and evaluation (GRADE).approach was used to evaluate the quality of our study from https://gdt.gradepro.org website.

Risk of bias (quality) assessment

The quality of included studies were evaluated with the Newcastle-Ottawa scale. The standards implemented by the Oxford Centre for Evidence-based Medicine were applied to assess the level of evidence included in the study.

PLANNED DATA SYNTHESIS

Strategy for data synthesis

The Review Manager 5.3, Stata 16, and MetaXL software were used for the statistical analysis. Statistical analysis using Stata 15.0 software. Significance was combined with OR / RR and the corresponding 95% CI and significance was determined by Z-test. Heheterogeneity of the included literature was analyzed by Q test and I 2, when P <0.05, I 2> 50% indicating high heterogeneity, using random effects model and instead fixed effects model (Higgins et al., 2003). Sensitivity analysis was used to test the stability of the results and also by gender, sample size, index grouping, Hyperuricemia criteria, and quality assessment Subgroup analysis such as classification and sleep cycle was used to explore the influence of study characteristics on outcome variables and the causes of heterogeneity. Egger's test and Begg's rank correlation test were used to judge publication bias. If there was publication bias, non-parametric shear supplement method was used to correct the effect value

Analysis of subgroups or subsets

sex and age

REVIEW AFFILIATION, FUNDING AND PEER REVIEW

Review team members

Mr LUO Chun. ZHEJIANG CHINESE MEDCINE UNIVERSITY. China.

No conflict of interest declared.

Dr YU Xiali. Zhejiang Hospital of Traditional Chinese Medicine. China.

No conflict of interest declared.

Miss ZHANG Fengqi. ZHEJIANG CHINESE MEDCINE UNIVERSITY. China.

No conflict of interest declared.

Miss SHEN Danqian. School of Basic Medical Sciences, Zhejiang Chinese Medical University, Hangzhou, China. China.

No conflict of interest declared.

Professor XIE Zhijun (review guarantor). School of Basic Medical Sciences, Zhejiang Chinese Medical University, Hangzhou, China. China.

No conflict of interest declared.

Dr Sun Jing. The Second School of Clinical Medicine, Zhejiang Chinese Medical University, Hangzhou, China. China.

No conflict of interest declared.

Named contact

Dr YU Xiali (yuxialiyuxiali@163.com). Zhejiang Hospital of Traditional Chinese Medicine. China.

Review affiliation

ZHEJIANG CHINESE MEDCINE UNIVERSITY

Funding source

Additional non-commercial funding information
GZS2020024

Named contact

Luo Chun. China
doclc1998@163.com

TIMELINE OF THE REVIEW

Review timeline

Start date: 1 May 2024. End date: 18 June 2024.

Date of first submission to PROSPERO

05 March 2024

Date of registration in PROSPERO

16 March 2024

CURRENT REVIEW STAGE

Publication of review results

Results of the review will be published in English.

Stage of the review at this submission

Review stage	Started	Completed
Pilot work	✓	✓
Formal searching/study identification	✓	✓
Screening search results against inclusion criteria	✓	✓
Data extraction or receipt of IPD	✓	✓

Risk of bias/quality assessment	✓	✓
Data synthesis	✓	✓

Review status

The review is completed.

ADDITIONAL INFORMATION

PROSPERO version history

- Version 1.1, published 16 Mar 2024
- Version 1.0, published 16 Mar 2024

Review conflict of interest

Declared individual interests are recorded under team member details.. No additional interests are recorded for this review.

Country

China; United States of America; England; Korea, Republic of

Medical Subject Headings

Humans; Hyperuricemia; Incidence; Metabolic Syndrome; Risk Factors; Sleep; Sleep Duration; Sleep Quality; Uric Acid

Disclaimer

The content of this record displays the information provided by the review team. PROSPERO does not peer review registration records or endorse their content.

PROSPERO accepts and posts the information provided in good faith; responsibility for record content rests with the review team. The guarantor for this record has affirmed that the information provided is truthful and that they understand that deliberate provision of inaccurate information may be construed as scientific misconduct.

PROSPERO does not accept any liability for the content provided in this record or for its use. Readers use the information provided in this record at their own risk.

Any enquiries about the record should be referred to the named review contact
