# Supplementary material for: Nighttime sleep duration and the prevalence of hyperuricemia: a systematic review and network meta-analysis
Source: Front Neurosci. 2025 May 9;19:1436116. doi: 10.3389/fnins.2025.1436116 (PMC12098448; doi:10.3389/fnins.2025.1436116)
Supplement: Supplementary file 2 [file Supplementary_file_2.pdf]

Supplementary File 2：Search Strategy Table in each Data Base

Data Base	Search strategies
PubMed	(duration, sleep) OR (total sleep time) OR (sleep quantity) OR (quantity, sleep) OR (sleep quantities) OR (longitudinal sleep) OR (sleep insufficiency) OR (poor sleep) OR (sleep-deprived) OR (sleep problems) OR (sleep disturbances) OR (sleep efficiency) OR (sleep latency) OR (sleep disorders) OR (somnipathy) OR (sleep)[Mesh] AND (uric acid) OR (hyperuricemia) [Mesh]
Cochrane Library	#1<br>"duration, sleep" or " total sleep time" or "sleep quantity" or "quantity, sleep" or "sleep quantities" or "longitudinal sleep "or "sleep insufficiency" or "poor sleep" or "sleep-deprived" or "sleep problems" or "sleep disturbances" or "sleep efficiency" or "sleep latency" or "sleep disorders "or "somnipathy" or "sleep"<br>#2<br>"uric acid" OR "hyperuricemia"<br>#1 and #2
EMBASE	#1<br>"duration, sleep" or " total sleep time" or "sleep quantity" or "quantity, sleep" or "sleep quantities" or "longitudinal sleep "or "sleep insufficiency" or "poor sleep" or "sleep-deprived" or "sleep problems" or "sleep disturbances" or "sleep efficiency" or "sleep latency" or "sleep disorders "or "somnipathy" or "sleep"<br>#2<br>"uric acid" OR "hyperuricemia"<br>#1 and #2
CNKI	(duration, sleep+total sleep time+leep quantity+quantity, sleep+sleep quantities+longitudinal sleep+sleep insufficiency+poor sleep+sleep-deprived+sleep problems+sleep disturbances+sleep efficiency+sleep latency+sleep disorders+somnipathy+sleep) * (uric acid+hyperuricemia)
WANFANG	("duration, sleep"OR"total sleep time"OR"leep quantity"OR"quantity, sleep" OR "sleep quantities" OR "longitudinal sleep" OR "sleep insufficiency" OR "poor sleep" OR "sleep-deprived" OR "sleep problems" OR "sleep disturbances" OR "sleep efficiency" OR "sleep latency" OR "sleep disorders" OR "somnipathy" OR "sleep")AND ("uric acid" OR "hyperuricemia")
CBM	#1<br>"duration, sleep" or " total sleep time" or "sleep quantity" or "quantity, sleep" or "sleep quantities" or "longitudinal sleep "or "sleep insufficiency" or "poor sleep" or "sleep-deprived" or "sleep problems" or "sleep disturbances" or "sleep efficiency" or "sleep latency" or

	“sleep disorders ”or “somnipathy” or “sleep”<br>#2<br>“uric acid” OR “hyperuricemia”<br>#1 and #2
ClinicalTrials.gov	#1<br>“duration, sleep” or “ total sleep time” or “sleep quantity” or “quantity, sleep” or “sleep quantities” or “longitudinal sleep ”or “sleep insufficiency” or “poor sleep” or “sleep-deprived” or “sleep problems” or “sleep disturbances” or “sleep efficiency” or “sleep latency” or “sleep disorders ”or “somnipathy” or “sleep”<br>#2<br>“uric acid” OR “hyperuricemia”<br>#1 and #2
